# Development of Infrared Transmission Flame-Retardant Polyethylene Melt Blends and Melt-Blown Nonwovens

**DOI:** 10.3390/polym17212854

**Published:** 2025-10-26

**Authors:** Weizhu An, Yihui Wei, Youkuai Lin, Shihao Wang, Chengjian Li, Haiqian Yu, Xing Wu, Yinchao Zhu, Feichao Zhu, Munir Hussain

**Affiliations:** 1 College of Textile Science and Engineering, Zhejiang Sci-Tech University, Hangzhou 310018, Chinawsh2096@126.com (S.W.); 13456525825@163.com (C.L.); zyc87314678@163.com (Y.Z.); 2 Zhejiang Sci-Tech University Shengzhou Innovation Research Institute, Shengzhou 312400, China; 3 Huahao Nonwovens Co., Ltd., Longgang 325802, China; yklin@ehuahao.com

**Keywords:** polyethylene, melting index, melt-blown nonwoven, rheological properties, flame-retardant

## Abstract

Polyethylene (PE) melt-blown nonwoven materials exhibit excellent infrared transmission properties, making them well-suited for applications in infrared physiotherapy and smart building technologies. However, their high flammability and tendency to generate melting droplets and smoke seriously limit their applications. Herein, phosphorus-silicon flame-retardant PE melt-blown blends were prepared by the melt blending of ammonium polyphosphate (APP) and nano-silica (SiO_2_). Next, the thermal, rheological, and crystallization properties of the blends were investigated. Subsequently, flame-retardant PE melt-blown nonwoven materials were prepared and tested. It was found that APP and SiO_2_ decreased the melt flowability of the material, while slightly decreasing the melting point, increasing crystallinity and enhancing the thermal stability by shifting the decomposition temperature by 51 °C. Moreover, the presence of flame retardants increased the roughness and diameter of fibers. The limiting oxygen index (LOI) of the PE melt-blown materials with 10% APP and 1% SiO_2_ reached 28.6%, reaching the flame-retardant level without dripping during combustion. This highlights important guidelines for developing infrared-transmitting, flame-retardant PE nonwovens for safe and sustainable applications.

## 1. Introduction

Nowadays, energy management materials are very popular and important, among which polyethylene (PE) is a typical infrared transmission material. Melt blowing is a non-woven technology for the large-scale preparation of ultrafine fiber aggregates. Melt-blown nonwoven materials have the characteristics of a large specific surface area, high porosity, and low density [1], which can be widely used in filtration, heat preservation, sound absorption, medical, and other fields [2,3]. Due to its excellent aging resistance, electrical insulation, high thermal conductivity, and high infrared transparency, PE melt-blown materials have been developed for health, physiotherapy, and construction [4,5,6,7]. However, the LOI of PE is less than 18%, which is extremely flammable. The resulting droplets can cause secondary burns, which are harmful to human health and harm the ecosystem. PE melt-blown material is more flammable due to its small fiber diameter. Its application in infrared physiotherapy health care equipment, intelligent buildings, and other fields has been limited. Therefore, it is of great significance to develop PE infrared transmission materials that have flame-retardant properties. Currently, research on flame-retardant PE melt-blown materials is sparse, but many studies have been conducted on polyolefin blends [8,9]. The main ways to obtain flame-retardant properties of nonwoven materials are surface flame-retardant modification, such as impregnation [10], coating or deposition [11], blending, or copolymerization. The former has flame-retardant effects with poor stability, while the latter has durable flame-retardant performance and a simple production process, and its products are closer to the needs of consumers [12].

Halogen-free intumescent flame retardants (IFRs) are considered environmentally friendly because of their high efficiency and low environmental impact [13,14,15,16]. In a traditional IFR flame-retardant system, the quantity is large and the compatibility is poor; the flame-retardant effect is not long-lasting, while the performance of material processing is also poor [17]. Research publications on the flame retardancy of polyethylene systems during the last 10 years have been thoroughly evaluated. It was discussed how flame retardants from the phosphorus-based, melamine-based, nitrogen-based, inorganic hydroxide, boron-based, and silicon-based families affect the mechanical properties of PE, as well as their mechanisms and efficiency [18]. It is of great significance for the product development and market application of PE materials to find a new and efficient synergistic flame-retardant scheme for the IFR flame-retardant system and improve the water resistance of flame retardants. Ammonium polyphosphate (APP) [19] is a green flame retardant which is rich in phosphorus and nitrogen. The interface compatibility between APP and PE is poor without any treatment; however, after surface hydrophobic modification, the polarity of APP decreases, and the combination with PE improves.

In the combustion process of PE melt-blown fabric, APP does not produce toxic gases and plays a role in condensed-phase and gas-phase flame retardants, which are widely used in the flame-retardant treatment of PE. Nano-silica has the advantages of high strength, high toughness, and high stability [20]. It is also commonly used as an anti-dripping flame retardant for PE. The flame-retardant mechanism of nano-SiO_2_ can be explained as a condensed-phase flame retardant [21].

A study comprehensively evaluated the effect of introducing low-cost inorganic fillers such as copper slag (CS), basalt powder (BP), and expanded vermiculite (VM) into flame-retardant ammonium polyphosphate polyethylene composite (PE/APP). The cone calorimeter tests showed a cooperative effect between APP and VM, reducing the peak heat release rate (PHRR) by 60% compared to unmodified PE [22]. Moreover, the flammability of polypropylene and its intumescent flame-retardant composites composed of ammonium polyphosphate (APP), pentaerythritol (PER), and SiO_2_ was investigated, and the cooperative effects and synergistic mechanism of SiO_2_ discussed in detail. SiO_2_ has a cooperative effect in the PP/APP/PER system, and the optimum addition amount is 3.5 wt.%. The introduction of SiO_2_ improved the flame retardancy and char strength of the PP/APP/PER [23].

In addition, fire retardancy of polymeric materials highlights that flame-retardant systems effectively inhibit the transfer of heat and oxygen between the flame and PE, facilitate carbonization, and form a protective char layer on the surface of the PE flame-retardant blends [24]. This layer acts as a barrier, impeding further thermal degradation and enhancing the flame-retardant properties of the PE. Concurrently, nano-SiO_2_ becomes embedded within the char layer, forming Si–O–P structures that enhance the oxidation resistance and thermal stability of the char layer, preventing secondary combustion and increasing the residual carbon content of the PE blends. The resulting high-performance char layer functions as an effective flame-retardant barrier, reducing heat transfer and suppressing the release of combustible gases, thereby interrupting the sustained combustion of PE [25]. The cooperative effect of the flame-retardant mechanism of the APP/SiO_2_ composite system improves the overall flame-retardant performance of PE flame-retardant blends.

Herein, we prepared the melt-blown PE blends that were used as the matrix; the hydrophobic nano-SiO_2_ was used as the silicon source in the IFR system, and APP was used as the gas source and acid source to form an efficient synergistic flame retardant of Si, N, and P to improve the flame-retardant performance of PE. The flame-retardant PE melt-blown nonwoven materials were further prepared by the melt blending of two flame retardants with PE blends, which provided technical support for the application of infrared transmission flame-retardant PE melt-blown materials in infrared physiotherapy equipment.

## 2. Materials and Methods

The high-melt-index PE masterbatches were prepared in the lab. Polyethylene wax (PEW) 40% as plasticizer and 0.5% dicumyl peroxide (DCP) were added to 59.5% linear low-density polyethylene (LLDPE) and mixed in a high-speed mixer for about 15 min. The mixture was melted and extruded by a co-rotating twin-screw extruder, cooled in a water bath, and cut into pellets by a cutting machine to prepare the high-melt-index PE masterbatch and was labeled as M with a melt index of 450 g/10 min (at 230 °C, and with 1.6 kg load). Ammonium polyphosphate (APP) with the model type TF-241 was purchased from Guangzhou Zhanpu Chemical Co., Ltd. (Guangzhou, China). The APP was obtained by surface modification of the silane coupling agent. Hydrophobic nano-SiO_2_ with a purity of 99.5% and a particle size of 20 ± 5 nm was gained from Hangzhou Mick Chemical Instrument Co., Ltd. (Hangzhou, China).

**Figure 1:**
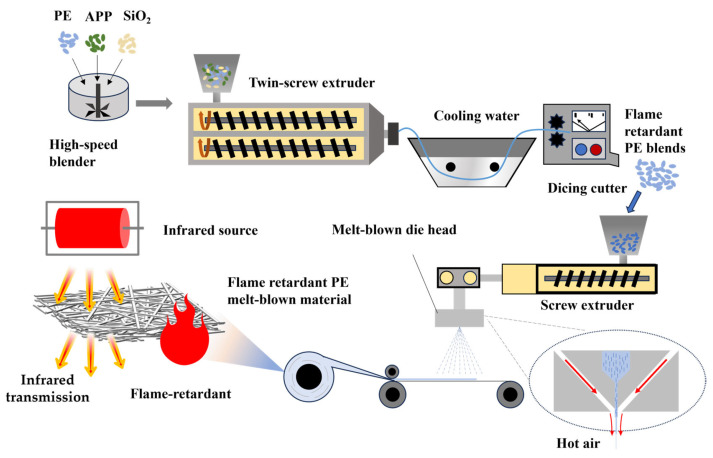
Schematic representation of flame-retardant PE blend and melt-blown material preparation methods.

Figure 1 is the preparation flow chart of the flame-retardant PE blends (FRM), and the components in the blending system are shown in Table 1. To improve the stability of the blends, it was dried at a constant temperature of 80 °C for 4 h in the oven, and the mixture was mixed in a blending machine for about 15 min according to the weight ratio of the scheme shown in Table 1. The samples were numbered FRM_1_, FRM_2_, FRM_3_, FRM_4_, FRM_5_, and FRM_6_, fully stirred evenly, and melt extruded through a co-rotating twin-screw extruder at 200 °C. After cooling in a water bath, it was cut into pellets by a cutting machine to prepare the flame-retardant PE melt-blown blends. The temperature parameters of the screw extruder are shown in Table 2. The screw speed was 150–200 r/min, and the cutting speed was 60–80 r/min.

Figure 1 shows the preparation flow chart of the flame-retardant PE melt-blown material (FRMB), which was prepared by an SJ-25 micro melt-blown tester (Zhangjiagang Arod Machinery Co., Ltd., Zhangjiagang, China). After extrusion, shear, and melt extrusion, the PE blends were stretched into micron/sub-micron fibers under the action of high-speed and high-temperature airflow on both sides of the spinneret and then received and wound by the receiving device. The prepared samples were labeled as FRMB_1_, FRMB_2_, FRMB_3_, FRMB_4_, FRMB_5_, and FRMB_6_. The PE melt-blown nonwoven material without flame-retardant modification was labeled as MB. Table 3 shows the process parameters of the melt-blown materials.

The melt indexes (MI) of the PE-modified blends were tested using the RL-Z1B1 melt flow rate meter (Shanghai Shangyan Scientific Instrument Co., Ltd., Shanghai, China). About 5 g of dried sample was taken, the test temperature was 190–260 °C, the load was 2.16 kg, and the average value was taken after 5 tests. The melt rheological properties of PE-modified blends at 230 °C were measured by an MCR301 rheometer (Anton Paar, Graz, Austria). The frequency scanning range was 0.1–100 rad/s.

A DSC 8000 differential scanning calorimeter (Perkin Elmer Company, Waltham, MA, USA) was used for thermal-crystallization performance testing. Samples of 5–6 mg were heated from 20 °C to 200 °C in a nitrogen atmosphere, with a heating rate of 10 °C/min, and maintained at 200 °C for 2 min. Then, the samples were cooled to 20 °C with a cooling rate of 10 °C/min, and the temperature was maintained for 2 min. After that, the samples were heated again to 200 °C, and the heating rate was 10 °C/min. The thermal stability of the PE-modified blends were tested using a 4000 thermogravimetric analyzer (TGA) (Perkin Elmer Company, USA) under nitrogen atmosphere protection. The test temperature was 25–600 °C, and the heating rate was 20 °C/min.

An Ultra 550 thermal field emission scanning electron microscope (FE-SEM, Germany Zeiss Optical Instrument Co., Ltd., Jena, Germany) was used to observe the surface morphology of flame-retardant PE melt-blown material and the material after combustion. Nano Measurer software (version 1.2) was used to process the scanning electron microscope image, 50 fibers in the electron microscope image were randomly selected to measure their diameter, and the diameter and its diameter distribution histogram were recorded.

For SEM analysis of post-combustion residues, samples were collected from the burned edge areas of the materials after the combustion tests, where the char layer formation was most evident. Loose droplets and fallen residues were removed, and the selected areas were gold-sputtered to enhance conductivity prior to imaging at higher magnifications.

The samples were cut into rectangles with a size of 5 cm × 2 cm, and the mechanical properties of the samples were tested using an Instron-3369 universal material testing machine (Instron company, Norwood, MA, USA). The cross-head speed was 50 mm/min. For each composition, three specimens (5 cm × 2 cm) were tested under identical conditions to ensure statistical reliability. The reported tensile strength and elongation values represent the average of five independent measurements, and the standard deviations were calculated to reflect experimental variability. The flame-retardant performance of the samples was tested using the CZF-3 vertical combustion instrument (Nanjing Jiangning District Analysis Instrument Factory, Nanjing, China). The flame retardancy of the samples was evaluated according to the GB/T 5455-2014 standard [26] (Vertical burning method). This standard specifies a vertical flame test especially for textiles to assess the burning behavior. In this method, a rectangular fabric specimen is mounted vertically and exposed to a controlled flame of 40 ± 2 mm under ambient conditions. The sample size was 60 mm × 200 mm and each sample was repeated 3 times. The test samples were dried in a 100° C oven for 30 min and then cooled before being tested under an atmosphere of 25 ° C and 40% humidity. The ignition time was three seconds, and the smoldering time and the duration of the remaining fire were recorded. The limiting oxygen index of the sample was evaluated by a JF-5 automatic oxygen index analyzer (Nanjing Jionglei Instrument Co., Ltd., Nanjing, China) and GB/T 5454-1997 standard [27]. The sample size was 150 mm × 60 mm. The oxygen concentration was adjusted, and the average value was taken 3 times.

## 3. Results and Discussion

### 3.1. Analysis of Melt Fluidity and Rheological Properties of Flame-Retardant Blends

**Figure 2:**
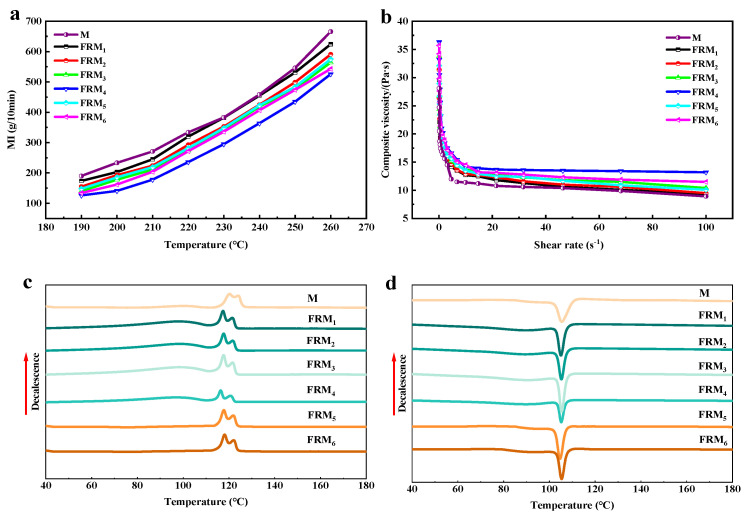
(**a**) Melt index curve and (**b**) rheological curve of pure and flame-retardant PE blends and DSC curve of flame-retardant PE blends: (**c**) heating and (**d**) cooling.

Figure 2a is the melt index curve of the flame-retardant PE melt-blown blends. It can be observed from the figure that the addition of APP and nano-SiO_2_ makes the melt flow performance of the material worse. The flame-retardant blends with 20% APP show a significant decrease in melt index. Because APP and SiO_2_ are inorganic particles, the addition of APP and SiO_2_ will increase the intermolecular force of PE and hinder the mutual movement of molecular chains, thus increasing the melt viscosity of flame-retardant blends and reducing the flow performance and MI [28]. Considering the actual situation that the blends will be yellowed by thermal oxidation at 260 °C, the processing temperature is still 230 °C. Figure 2b shows the melt rheological curves of PE blends tested at 230 °C. As shown in the curves of M and FRM_1_, FRM_2_, FRM_3_, and FRM_4_, with the increase in APP content, the viscosity of the system gradually increases at the same shear rate, which also reflects that the melt flow index of the blends gradually decreases and the difficulty of material extrusion increases. This effect is because APP plays a role in filling and strengthening the PE matrix, increasing the internal friction of the system, and hindering the movement of the molecular chain. According to the curves of FRM_2_, FRM_5,_ and FRM_6_, it can be seen that the addition of SiO_2_ will also increase the viscosity of the system at the same APP addition and shear rate, thus reducing the melt flow index of the PE blends. In addition, compared with APP, the addition of SiO_2_ has a more significant effect on the viscosity of the system. This may be because nano-silica has a smaller particle size and a larger specific surface area, and the interaction with the PE matrix is more significant. It will enhance the internal friction of the system through physical adsorption or chemical bonding, thus affecting the viscosity and the melt flow index.

### 3.2. Analysis of Thermal-Crystallization Performance of Flame-Retardant Blends

Figure 2c,d show the DSC curves of the flame-retardant PE blends, and the corresponding thermal-crystallization performance parameters are listed in Table 2. It can be seen from the figure that the melting point of the flame-retardant PE melt-blown blends decreased slightly after the addition of flame-retardant inorganic particles. The addition of APP and SiO_2_ nano-inorganic particles reduced the order of the molecular arrangement of the PE matrix. It can be seen from Figure 2d that the cool crystallization temperature (*T*_cc_) of the flame-retardant PE blends increased slightly. The supercooling rate of flame-retardant PE blends is reduced. This indicates that the crystallization rate of flame-retardant PE blends increases slightly. In addition, as shown in Table 4, with the increase in APP content, the crystallinity of flame-retardant PE melt-blown blends increased first and then decreased. The crystallinity of neat M is 21.7%. As an inorganic flame retardant, the surface of APP particles can be used as a heterogeneous nucleation site, which can significantly shorten the crystallization induction period of PE.

The polar surface of APP adsorbs the PE molecular chain, which reduces the nucleation activation energy and promotes the formation of a crystal nucleus. The low content of APP accelerates the initial stage of crystallization through heterogeneous nucleation, which increases the cooling crystallization temperature and accelerates the crystallization rate [29]. However, excessive APP will hinder the regular arrangement and movement of PE molecular chains, resulting in a decrease in the crystal growth rate. The crystallinity of sample FRM_3_ is 23.7%. When the amount of added APP reaches 20%, it is easy to agglomerate and hinder the crystallization of PE, making the crystallinity slightly worse. From the comparison results of FRM_2_ and FRM_5_, it can be concluded that the crystallinity of flame-retardant PE melt-blown blends increases with the increase of SiO_2_ content.

The surface adsorption of SiO_2_ is dominant, which preferentially fixes the PE molecular chain to form a locally ordered structure and accelerates nucleation. The polar surface of APP preferentially adsorbs PE chain segments to form initial nuclei, while SiO_2_ further provides nucleation sites through physical adsorption, and the two play a synergistic role to form multiple nucleation centers.

### 3.3. Analysis of Thermal Stability of Flame-Retardant Blends

**Figure 3:**
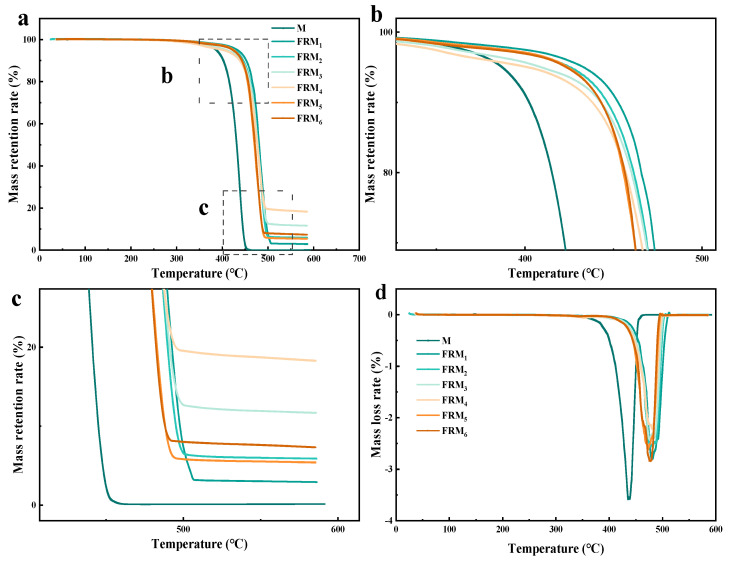
(**a**) TGA curves of pure and flame-retardant PE blends. Thermal cracking (**b**) begins and (**c**) ends in the TGA curves. (**d**) DTG curves of pure and flame-retardant PE blends.

The TGA and DTG curves of sample M in the high-melt-index blends and the six flame-retardant PE melt-blown blends are shown in Figure 3. It can be seen from Figure 3a that the thermal degradation temperature range of neat M and the six flame-retardant PE melt-blown blends is 350–500 °C. The complete degradation temperature of sample M is about 450 °C, and the mass retention rate is 0% when the degradation is complete, and the sample is completely decomposed. The corresponding temperatures of T_5wt%_, T_50wt%_, and T_95wt%_ of M were 392.6 °C, 433.1 °C, and 448.8 °C, respectively, while the corresponding temperatures of T_5wt%_, T_50wt%_, and T_95wt%_ of FRM_1_ blends with 5% APP were 443.8 °C, 481.0 °C, and 503.3 °C, respectively. As shown in Figure 3b,d, compared with sample M, the degradation temperature of FRM_6_ increased by about 51 °C, and APP and SiO_2_ delayed the thermal decomposition reaction of PE. T_5_%, T_50_%, and the char residue of neat M and the flame-retardant PE melt-blown blends from TGA analysis are shown in Table 5.

In addition, with the increase in APP content, the T_5wt%_ decomposition temperature of flame-retardant PE blends decreases, and the thermal decomposition rate increases, which is due to the thermal decomposition of APP. In this case, the early thermal decomposition of APP in PE composites is beneficial, producing non-combustible gas and reducing the concentration of combustible materials [30]. It helps APP and SiO_2_ to form an intumescent char layer on the surface of PE, and isolates the heat transfer between the external fire source and the matrix, thereby inhibiting the spread of fire and achieving a flame-retardant effect [31]. Adding 0.5% and 1% SiO_2_ based on 10% APP, the T_5wt%_ decomposition temperature of the material decreased slightly, and the T_50wt%_ decomposition temperature increased by about 24 °C, which indicates that SiO_2_ shows a good synergistic flame-retardant effect with APP.

As shown in Figure 3a,c, the carbon residue of samples FRM_1_, FRM_2_, FRM_3_, FRM_4_, FRM_5_, and FRM_6_ were 2.9%, 5.9%, 11.7%, 18.3%, 5.4%, and 7.3%, respectively. This phenomenon indicates that APP has a good catalytic effect on the formation of the char layer during PE combustion. From the analysis of FRM_2_ and FRM_5_ curves, it can be seen that the addition of a very small amount of SiO_2_ has little effect on the formation of the carbon layer of PE combustion, but it is worth noting that the complete thermal decomposition temperature of the system is significantly reduced after the addition of SiO_2_, which may be due to the addition of SiO_2_ to accelerate the carbonization reaction of PE molecular chain during thermal decomposition. According to the FRM_5_ and FRM_6_ curves, the addition of SiO_2_ can also increase the amount of carbon residue after complete thermal decomposition, which indicates that SiO_2_ can also promote the formation of a carbon layer.

Compared with other reported flame-retardant strategies, the APP ± SiO_2_ system used in this study shows a balanced improvement in thermal stability and char formation at relatively low additive loadings [32]. While conventional metal hydroxide or phosphorus systems require high contents that often impair mechanical properties, the APP–SiO_2_ formulation increased T_50_% to about 481 °C and produced up to 18.3% char residue, indicating strong condensed-phase protection. The early decomposition of APP slightly reduces T_5_%, but the addition of SiO_2_ enhances char cohesion and barrier integrity [33], demonstrating a clear synergistic flame-retardant effect with minimal processing drawbacks [34]. The DTG curves revealed single major degradation peaks for all samples, indicating one-step thermal decomposition behavior. The peak decomposition temperature shifted toward higher values for the APP–SiO_2_ blends compared to neat PE, confirming the enhanced thermal stability. Moreover, the reduced peak intensity in DTG curves suggests a slower degradation rate due to the protective char layer formed during combustion.

### 3.4. Analysis of Surface Morphology and Diameter of Flame-Retardant Melt-Blown Materials

**Figure 4:**
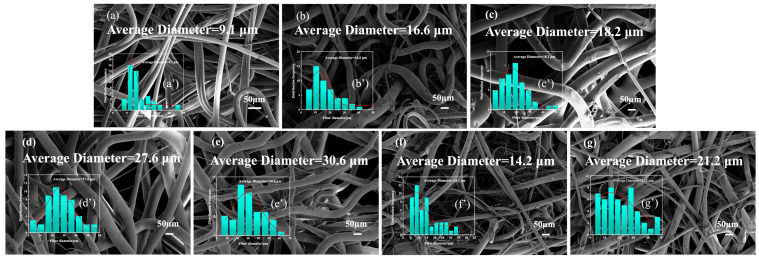
(**a**–**g**) SEM images and (**a’**–**g’**) fiber diameter distribution of flame-retardant PE melt-blown materials: (**a**(**a**’)) M, (**b**(**b’**)) FRMB_1_, (**c**(**c’**)) FRMB_2_, (**d**(**d’**)) FRMB_3_, (**e**(**e’**)) FRMB_4_, (**f**(**f’**)) FRMB_5_, (**g**(**g’**)) FRMB_6_. All SEM images were captured at a magnification corresponding to a scale bar of 50 μm.

Figure 4a–g are the SEM images of pure and flame-retardant PE melt-blown material, and Figure 4a′–g′ are the corresponding fiber diameter distributions. The dispersion of nano-particles in the material and the diameter of melt-blown fibers are important factors affecting the properties of melt-blown nonwovens. It is observed that the surface of PE melt-blown fiber is relatively smooth, and there is agglomeration on the surface after flame-retardant modification, and the roughness increases. The spinnability of flame-retardant PE blends is lower than that of unmodified high-melt-index PE blends. The average fiber diameter of the neat MB without flame-retardant modification was 9.1 μm, and the diameter distribution range of the fiber was 3.0–21.0 μm. With the increase in APP and SiO_2_ content, the average diameter of the fiber increased. The average diameter of the sample FRMB_1_ reached 16.6 μm, and the average diameter of the sample FRMB_4_ reached 30.6 μm. It shows that when the mass fraction of the flame-retardant is too high, the fiber is difficult to fully stretch, the fiber diameter becomes thicker, and the fibers have difficulty crossing each other. The hot melt bonding of the points is consolidated into a fiber network. FRMB_4_ shows that when the pores between the fibers are too large, the strength of the fiber net will also be affected, and the overall stability of the flame-retardant PE melt-blown material will decrease.

### 3.5. Analysis of the Mechanical Properties of Flame-Retardant Melt-Blown Materials

**Figure 5:**
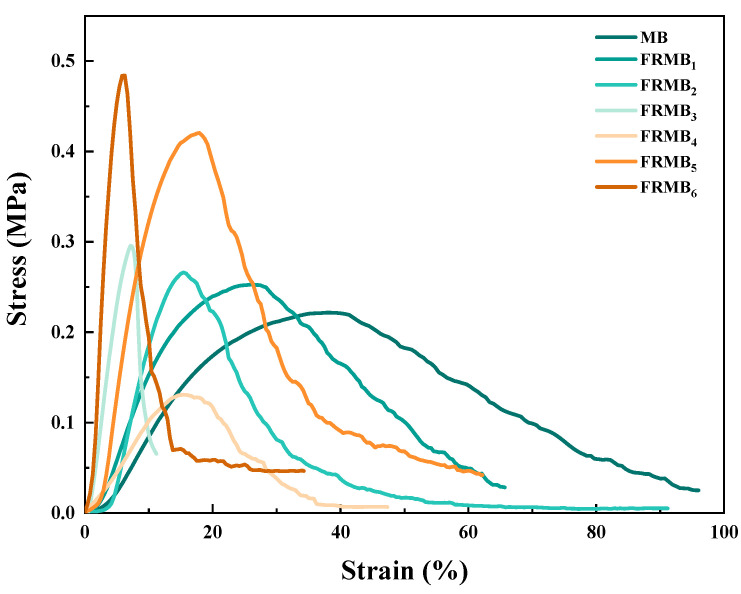
The stress–strain curve of pure and flame-retardant PE melt-blown materials.

Figure 5 shows the stress–strain curves of flame-retardant PE melt-blown materials. The stress–strain curves represent the average data from three replicates per composition. By comparing MB with FRMB_1_, FRMB_2_, and FRMB_3_, the tensile strength of the materials increases with the increase in APP content. This deviates from the general situation where the addition of APP weakens the tensile strength, which may be due to the special combination of the high-melt-index PE used by us and APP. APP can act as a reinforcing phase in PE, thereby improving its tensile strength to a certain extent. However, when the APP addition is too high, the tensile properties of the material will be greatly reduced. By comparing MB with FRMB_5_ and FRMB_6_, it can be seen that the addition of nano-SiO_2_ can improve the tensile strength of the material. This is because nano-SiO_2_ has a high specific surface area and surface energy, which can form a good interfacial bond with the PE matrix, thereby effectively dispersing stress. The addition of both APP and SiO_2_ will reduce the elongation at the break of the PE melt-blown material because both will restrict the movement of PE molecular chains, thereby reducing the toughness of the material.

### 3.6. Analysis of the Flame-Retardant Performance of Flame-Retardant Melt-Blown Materials

**Figure 6:**
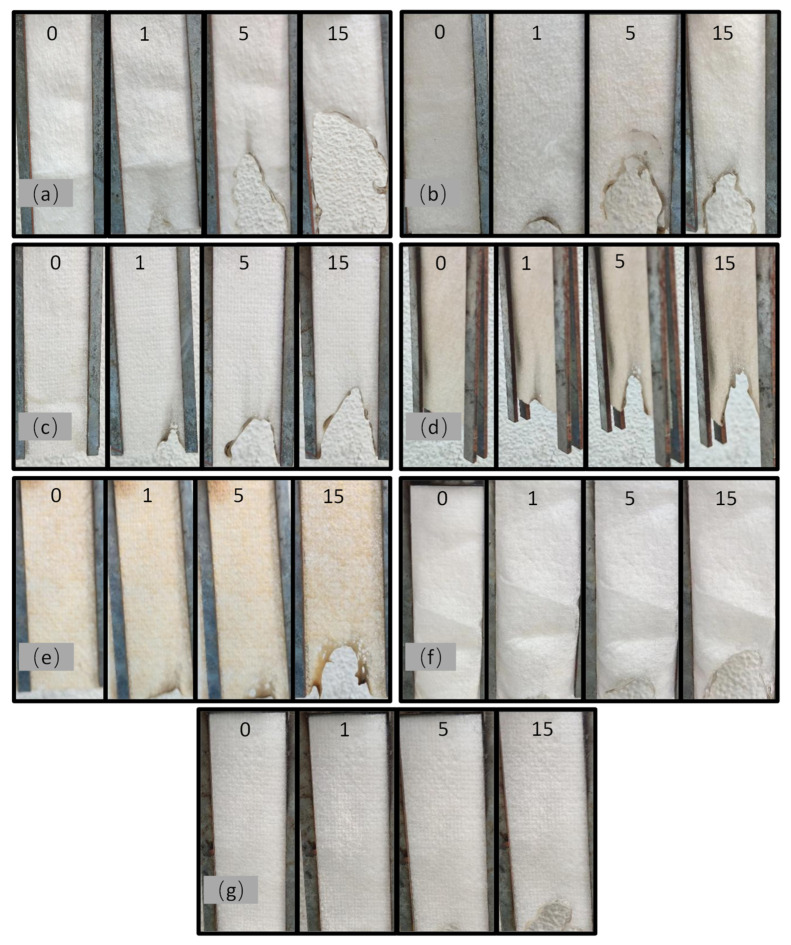
The combustion process of samples (**a**) MB, (**b**) FRMB_1,_ (**c**) FRMB_2_, (**d**) FRMB_3_, (**e**) FRMB_4_, (**f**) FRMB_5_, and (**g**) FRMB_6_.

Figure 6 shows the combustion process of neat MB and 6 kinds of flame-retardant PE melt-blown materials. The relevant data are listed in Table 6.

It can be seen from Figure 6a that when neat MB was affected by the heat source, a droplet-associated flame was generated, and the sample was seriously damaged. When the droplets encountered combustibles, they could cause secondary combustion, which indicates that this MB is an extremely flammable material. As shown in Figure 6b, when the FRMB_1_ was burned, the material melted and slowly burned during the combustion process, with slight amounts of white smoke and a little black residue. The after-burning time was shortened from 3.37 s to 1.34 s, and the smoldering time of the material was 0 s. FRMB_1_ has obvious flame-retardant properties during the combustion process. In Figure 6c, the material melted, and there was no flame combustion. White smoke and black residues were generated, and the smoldering and afterburning times were 0 s. It had the effect of self-extinguishing, but there were droplets. During the combustion process of sample FRMB_3_, shown in Figure 6d, the material still shrank, but there was no flame combustion. White smoke and black residues were generated, but no droplets were generated. In the combustion process of sample FRMB_4_, shown in Figure 6e, the sample melted and shrank because of the fire, with no flame combustion. More white smoke and black residue were produced, and no droplet phenomenon occurred.

As shown in Figure 6f, during the combustion process of FRMB_5_, there was melting shrinkage and no flame combustion. White smoke, black residues, and droplets were produced. According to Figure 6g, during the combustion process of sample FRMB_6_, there was melting-induced shrinkage and no flame combustion, producing white smoke and black residue, but no droplets. Combined with thermal performance analysis, the enhanced char yield and thermal stability can be attributed to the cooperative interaction between the phosphorus- and silicon-containing components, which promote the formation of a protective layer that inhibits heat and mass transfer during combustion. APP promotes the formation of the char layer, while SiO_2_ stabilizes the char layer. APP decomposes at high temperatures to produce strong acids such as phosphoric acid and polyphosphoric acid, which catalyze the dehydration and crosslinking of the matrix material PE to form an expanded char layer. This process significantly reduces the release of flammable volatiles by promoting condensed phase flame retardancy. Mesoporous SiO_2_ can be embedded in the char layer as a skeleton, and a Si-O-P bond is formed, which can improve its oxidation resistance and mechanical strength and prevent the char layer from disintegrating. When the concentration of SiO_2_ increases, the area of the char layer formed outside increases, which proves that there is a flame-retardant synergy between SiO_2_ and APP, and the flame-retardant effect is obvious, which improves the safety of the material. Gas-phase flame-retardant combined with condensed-phase flame-retardant effectively improves the flame-retardant performance of flame-retardant PE melt-blown materials.

**Figure 7:**
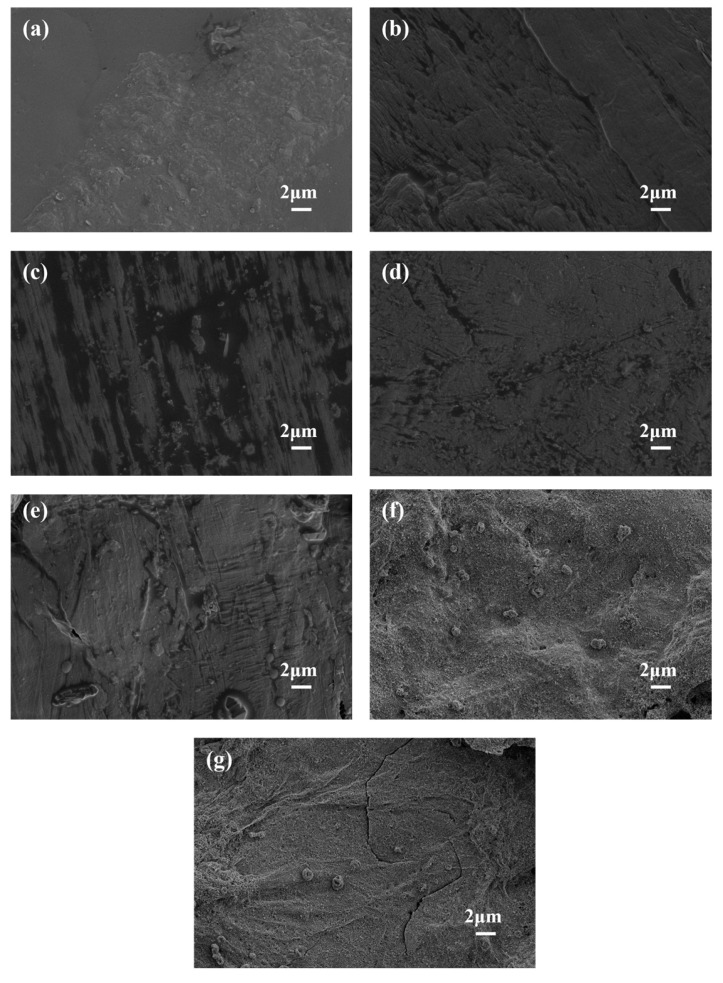
The SEM images of the residual carbon of samples (**a**) MB, (**b**) FRMB_1_ (**c**) FRMB_2_, (**d**) FRMB_3_, (**e**) FRMB_4_, (**f**) FRMB_5_, and (**g**) FRMB_6_. All SEM images were captured at a magnification corresponding to a scale bar of 2 μm.

Figure 7 is the SEM image of the residual carbon of sample MB and six flame-retardant PE melt-blown materials. It can be seen from Figure 7a that after the combustion reaction of neat MB, the material molecules decomposed rapidly, and the carbon content of the material remained low because the macromolecular chain of the material does not contain flame-retardant components. According to Figure 7b–g, the sample after flame-retardant treatment formed a dense char layer during combustion, which played a role in protecting PE and effectively inhibiting the release of combustible gas. From the SEM images of the low magnification version of Figure 7f,g, it can be seen that a uniform and dense char layer was formed on the surface of the material. The flame-retardant PE melt-blown nonwoven materials FRMB_3_, FRMB_4_, and FRMB_6_ did not form droplets during the combustion process because sufficient APP and nano-SiO_2_ can effectively adsorb and fix combustibles. The co-deposition of APP and SiO_2_ is more likely to form a dense char layer during PE combustion, which makes PE less volatile and releases and enhances the thermal stability of the material. Quantitative analysis from TGA (Table 5) complements these SEM observations, showing significantly higher char residue yields in flame-retardant samples (e.g., 10–15% at 800 °C for FRMB4–FRMB6) compared to near 0% for neat MB, confirming the formation of a dense, protective char layer that impedes further decomposition.

**Figure 8:**
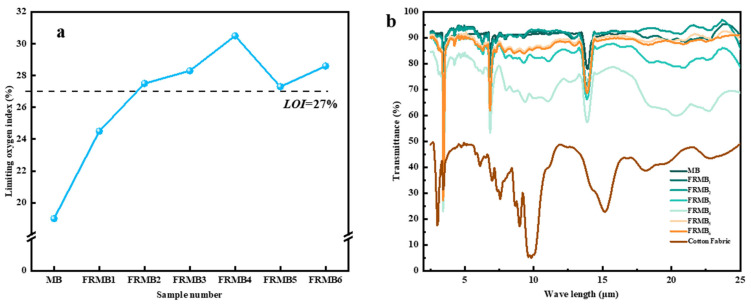
(**a**) The limiting oxygen index and (**b**) infrared transmittance curves of flame-retardant PE melt-blown materials.

Figure 8a is the limiting oxygen index curve of neat MB and six flame-retardant PE melt-blown materials. It can be seen that the limiting oxygen index of neat MB is 19%, which indicates that it is a flammable material. The limiting oxygen index of the material after flame-retardant treatment increases. As the APP mass fraction increased to 10%, the limiting oxygen index of FRMB_2_ reached 27.5%, reaching the flame-retardant level. The limiting oxygen index of sample FRMB_4_ reached 30.5%. However, due to the high content of APP, the viscosity of the material increased, and the difficulty of material processing increased. The limiting oxygen index of samples FRMB_5_ and FRMB_6_ with an APP addition of only 10% was 27.3% and 28.6%, respectively, which also reached the flame-retardant level. This is because the silicon hydroxyl group on the surface of SiO_2_ condensed with the polyphosphoric acid produced by APP decomposition to form a Si-O-P covalent network, which significantly improved the thermal stability of the char layer.

### 3.7. Infrared Performance Analysis of Flame-Retardant Melt-Blown Materials

Figure 8b is the infrared transmittance of PE nonwovens and cotton fabric. The 5-micron wavelength infrared light transmittance of flame-retardant melt blown materials prepared by MB, FRMB_1_, FRMB_2_, FRMB_3_, FRMB_4_, FRMB_5_, FRMB_6,_ and cotton fabrics were 91.4%, 93.8%, 93.5%, 80.8%, 83.7%, 91.3%, 90.3%, and 48.8%, respectively. The infrared transmittance of PE nonwovens is higher than that of cotton fabric. With the increase in APP mass fractions, the infrared transmittance of the materials decreased; the transmittance of other wavelengths of infrared light also conforms to this rule. APP is an inorganic salt that is insoluble in organic polymers such as PE, resulting in phase separation and the formation of a scattering center. As infrared light passes through a material, these scattering points deflect the light from its original path, reducing transmission and increasing haze, thus resulting in a decrease in the infrared transmittance of the material. The infrared transmittance of the FRMB_5_ and FRMB_6_ samples was basically the same as that of the FRMB_1_ sample. This is because the particle size of nano-SiO_2_ is much smaller than the wavelength of infrared light. According to the Rayleigh scattering theory, the scattering effect will be very small, so it will not significantly affect the infrared transmittance and will achieve a balance between flame retardant and infrared transmission.

**Figure 9:**
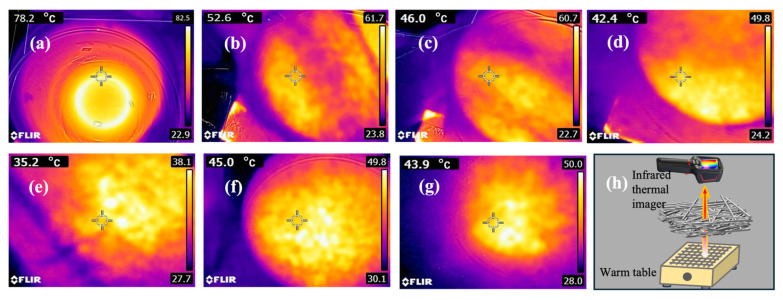
Infrared thermal images obtained by thermal imagers of samples (**a**) MB, (**b**) FRMB_1_, (**c**) FRMB_2_, (**d**) FRMB_3_, (**e**) FRMB_4_, (**f**) FRMB_5_, (**g**) FRMB_6_, and (**h**) infrared thermal imaging schematic diagram.

Figure 9 shows the infrared thermal imaging images of different materials obtained by a thermal imager and the infrared thermal imaging schematic diagram. The transmission temperatures of the flame-retardant melt-blown materials prepared from the samples MB, FRMB_1_, FRMB_2_, FRMB_3_, FRMB_4_, FRMB_5_, and FRMB_6_ were 78.2 °C, 52.6 °C, 46.0 °C, 42.4 °C, 35.2 °C, 45 °C, and 43.9 °C, respectively. Under the same conditions, the permeation temperature of FRMB_1_ (52.2 °C) was slightly higher, which may be due to the reduction in the thickness of FRMB_1_. The permeation temperature is related to the material thickness; the thicker the material, the lower the corresponding permeation temperature. The permeation temperatures of the flame-retardant PE nonwoven materials were all lower than those of neat MB, and the permeation temperature of the materials decreased with the increase in the mass fractions of APP and SiO_2_.

## 4. Conclusions

In conclusion, APP/SiO_2_ flame-retardant PE blends were prepared by melt blending, and corresponding PE materials were prepared by melt blowing. The melt fluidity, rheological properties, thermal crystallization properties, and thermal stability of flame-retardant PE blends were analyzed and compared. Adding an appropriate amount of APP and nano-SiO_2_ to the PE blends reduced its melt-blown machinability but increased the crystallinity of PE and significantly improved its thermal stability. Among the prepared melt-blown materials, the flame-retardant PE melt-blown material with 10% APP and 1% nano-SiO_2_ (FRMB_6_) had the best comprehensive performance. The limiting oxygen index of FRMB_6_ was 28.6%, which reached an excellent flame-retardant level. FRMB_6_ did not drip during the combustion process, which ensures the combustion safety of the material. This can also be attributed to the cooperative effect of APP and SiO_2_. APP decomposed at high temperature to produce phosphoric acid or polyphosphoric acid to catalyze the dehydration and crosslinking of PE, and also produced NH_3_ and other gases to promote the formation of an expanded char layer. SiO_2_ reacted with phosphoric acid or polyphosphoric acid to form a Si-O-P bond, which enhanced the stability of the char layer. In addition, FRMB_6_ still maintained a high infrared transmittance compared to the pure PE melt-blown material. This research establishes an important reference and strategy for the development of infrared transmission flame-retardant polyethylene nonwoven fabrics.

## Figures and Tables

**Table 1:** The mass ratio of each component of the experimental samples.

Samples Label	Mass Ratio of M (%)	Mass Ratio of APP (%)	Mass Ratio of SiO_2_ (%)
FRM_1_	95	5	0
FRM_2_	90	10	0
FRM_3_	85	15	0
FRM_4_	80	20	0
FRM_5_	89.5	10	0.5
FRM_6_	89	10	1

**Table 2:** Temperature parameters of each zone of the screw extruder.

Zone	First Zone	Second Zone	Third Zone	Fourth Zone	Fifth Zone	Material
Temperature/°C	180	190	195	200	200	180

**Table 3:** Melt-blown material processing parameters.

	First Zone	Second Zone	Third Zone	Fourth Zone	Die	Hot Air	Spinneret Hole	Pick-Up Range
Parameters	180 °C	210 °C	230 °C	230 °C	230 °C	230 °C	0.5 mm	20 cm

**Table 4:** DSC parameters (cooling crystallization temperature (*T*_cc_), cooling crystallization enthalpy (Δ*H_cc_*), melting temperature (*T*_m_), melting enthalpy (Δ*H_m_*), and crystallinity (*X*_c_)) of flame-retardant PE blends.

Sample Labels	*T*_cc_ (°C)	Δ*H_cc_* (J/g)	*T*_m_ (°C)	Δ*H_m_* (J/g)	*X*_c_ (%)
M	104.7 ± 0.3	38.2 ± 1.2	120.2 ± 0.4	44.6 ± 1.4	21.7 ± 0.5
FRM_1_	105.2 ± 0.3	53.5 ± 1.5	118.3 ± 0.4	45.3 ± 1.5	22.3 ± 0.5
FRM_2_	105.3 ± 0.3	55.1 ± 1.6	118.0 ± 0.3	47.2 ± 1.6	22.6 ± 0.5
FRM_3_	105.8 ± 0.4	73.3 ± 2.1	117.7 ± 0.3	52.5 ± 1.7	23.7 ± 0.6
FRM_4_	105.7 ± 0.3	36.4 ± 1.1	116.1 ± 0.3	38.4 ± 1.3	23.4 ± 0.6
FRM_5_	105.8 ± 0.4	70.9 ± 2.0	117.9 ± 0.4	56.7 ± 1.8	22.8 ± 0.5
FRM_6_	106.1 ± 0.4	72.1 ± 2.2	118.8 ± 0.3	58.1 ± 1.9	23.2 ± 0.6

**Table 5:** T_5_%, T_50_%, and char residue of neat M and flame-retardant PE melt-blown blends from TGA analysis.

Sample Labels	T_5%_ (°C)	T_50%_ (°C)	Char Residue (°C)
M	392.6 ± 1.2	433.1 ± 1.5	0.1 ± 0.05
FRM_1_	443.8 ± 1.1	481.0 ± 1.3	2.9 ± 0.2
FRM_2_	438.3 ± 1.0	477.6 ± 1.4	5.9 ± 0.3
FRM_3_	409.5 ± 1.2	477.6 ± 1.3	11.7 ± 0.3
FRM_4_	401.5 ± 1.1	476.5 ± 1.2	18.3 ± 0.4
FRM_5_	424.0 ± 1.2	470.9 ± 1.5	5.4 ± 0.3
FRM_6_	423.4 ± 1.1	471.1 ± 1.4	7.3 ± 0.3

**Table 6:** The combustion behavior of sample MB and six kinds of flame-retardant PE melt-blown materials.

Sample Number	Flame Duration (s)	Afterglow Time (s)	Droplet Generation
MB	3.73	0	Yes
FRMB_1_	1.34	0	Yes
FRMB_2_	0	0	Yes
FRMB_3_	0	0	no
FRMB_4_	0	0	no
FRMB_5_	0	0	yes
FRMB_6_	0	0	no

## Data Availability

The original contributions presented in this study are included in the article. Further inquiries can be directed to the corresponding authors.
